# Association between intraoperative hypotension and adverse clinical outcomes after esophagectomy for esophageal cancer: retrospective observational study

**DOI:** 10.1186/s40981-025-00826-4

**Published:** 2025-10-31

**Authors:** Takashi Juri, Koichi Suehiro, Hikaru Yasuhara, Masayo Takai, Shinta Yasuda, Aya Kimura, Kanae Takahashi, Yohei Fujimoto, Takashi Mori

**Affiliations:** 1 Department of Anesthesiology, Osaka Metropolitan University Graduate School of Medicine, Osaka, Japan; 2 Department of Medical Statistics, Osaka Metropolitan University Graduate School of Medicine, Osaka, Japan

**Keywords:** Blood pressure, Hypotension, Esophagectomy

## Abstract

**Background:**

Esophagectomy for esophageal cancer is complex and frequently complicated by pulmonary issues, cardiac problems, and anastomotic leakage. While numerous factors contribute to these complications, the specific effects of intraoperative hypotension (IOH) on postoperative outcomes remain poorly understood. This study examined the relationship between IOH severity during esophagectomy and subsequent complications.

**Methods:**

This retrospective observational study analyzed patients undergoing elective open transthoracic or thoraco-laparoscopic esophagectomy for esophageal cancer between May 2007 and February 2020. Multiple logistic regression assessed IOH’s association with primary outcomes (composite of major complications including 30-day mortality, reoperation, anastomotic leakage, pneumonia, reintubation, and prolonged ventilation > 48 h) and secondary outcomes of anastomotic leakage. Additionally, Cox regression analyzed IOH’s impact on long-term prognosis.

**Results:**

The final cohort comprised 884 patients. The most prevalent postoperative complications were anastomotic leakage (25.2%) and pneumonia (23.4%). The 30-day mortality rate was 0.8%. No significant association existed between IOH and major composite outcomes across mean blood pressure (MBP) and systolic blood pressure thresholds. However, IOH was significantly associated with anastomotic leakage when MBP fell below 65 mmHg for extended periods (adjusted odds ratio: 1.02 per 10-min interval, 95% confidence interval: 1.01–1.04, *P* = 0.01). IOH did not significantly affect long-term survival.

**Conclusions:**

This study did not identify a significant association between intraoperative hypotension and composite major postoperative complications or long-term survival outcomes. However, intraoperative hypotension, specifically prolonged episodes with mean blood pressure below 65 mmHg, was significantly associated with the short-term complication of anastomotic leakage.

**Trial registration:**

UMIN Clinical Trials Registry, UMIN000040455. Registered 28 May 2020, https://center6.umin.ac.jp/cgi-open-bin/ctr/ctr_view.cgi?recptno=R000046165.

## Background

Intraoperative hypotension (IOH) has been extensively studied and has been linked with several adverse outcomes, including acute kidney injury (AKI), myocardial injury, and increased mortality rates [1–3]. Despite the substantial body of evidence highlighting these associations, a conclusive definition of IOH remains elusive. Various thresholds have been proposed to optimize patient outcomes; however, the search for an optimal threshold is continuing [4]. Both the absolute minimum arterial blood pressure (BP) and the area under the BP threshold are strongly associated with organ dysfunction [5]. Recent research has suggested a plausible link between IOH and adverse long-term postoperative outcomes [6].

Esophagectomy, a pivotal treatment for potentially curable esophageal cancer, remains one of the most technically demanding surgical procedures with high rates of postoperative complications despite advancements in surgical techniques and perioperative care [7]. Postoperative complications following esophagectomy include pulmonary and cardiac complications, as well as anastomotic leakage, a hallmark complication of this procedure [8]. Numerous factors have been implicated as potential contributors to these complications, including the size of the surgical facility, blood transfusion, infusion volume, and preoperative comorbidities [9–12]. However, the specific effect of IOH during esophagectomy on postoperative outcomes remains unclear [13, 14]. Although the detrimental effects of IOH have been demonstrated in various surgeries, its specific role in esophagectomy warrants further exploration.

Given the limited understanding of the role of IOH in esophagectomy-related postoperative complications, we hypothesized that IOH is associated with these complications and that this association intensifies with increasing severity of hypotension. Therefore, this study aimed to investigate the relationship between IOH and adverse clinical outcomes after esophagectomy.

## Methods

### Study design

This retrospective, observational study was approved by the Ethics Committee of our hospital (No.2019–108) and registered with the UMIN Clinical Trials Registry (UMIN000040455). The requirement for written informed consent was waived because all protected personal information was anonymized prior to analysis.

### Inclusion criteria

Patients who underwent elective open transthoracic or thoraco-laparoscopic esophagectomy for esophageal cancer at our hospital between May 2007 and February 2020 were included in this study.

### Exclusion criteria

We excluded patients who underwent complex and prolonged surgical procedures, which were defined as any of the following: (1) free jejunal reconstruction with vascular anastomosis, (2) esophagectomy combined with resection of other organs (e.g., cholecystectomy, hepatectomy, or pulmonary resection), and (3) two-stage surgeries.

### Patients

After applying the predefined inclusion and exclusion criteria, a total of 884 patients were included in the final analysis. All esophagectomies were performed using a McKeown (three-incision) approach with a cervical anastomosis. The gastric conduit was reconstructed almost exclusively through the posterior mediastinal route, with very few cases using the retrosternal route. No robot-assisted procedures were undertaken during the study period. An intravenous bolus of methylprednisolone (500 mg) was administered at the initiation of surgery in all patients, regardless of body weight.

### Hemodynamic data collection

Intraoperative hemodynamic data were collected from electronic anesthesia records. Only invasive BP measurements obtained from radial artery catheters were analyzed, as these are considered more accurate [15]. Non-invasive BP measurements obtained using the oscillometric method were excluded from the analysis. Invasive BP data were extracted at 1-min intervals, and artifacts were removed using the following algorithm, adapted from previous studies: (1) systolic blood pressure (SBP) readings exceeding 300 mmHg or falling below 20 mmHg, (2) diastolic blood pressure (DBP) readings exceeding 225 mmHg or below 5 mmHg, and (3) SBP readings less than 5 mmHg above the DBP [16, 17].

### Definition of hypotension

Thresholds were established at mean blood pressures (MBP) of 55, 60, and 65 mmHg, as well as SBP of 70, 80, and 90 mmHg. For each threshold, we quantified the burden of hypotension by calculating the time spent below the threshold and deriving both the area under the threshold (AUT) and the time-weighted average (TWA). AUT—representing the cumulative hypotensive burden—was computed by a rectangular (step-function) method, summing across successive intervals the product of the interval length and the difference between the threshold and the corresponding subthreshold BP value; TWA was defined as AUT divided by the total duration of invasive arterial pressure monitoring, thereby standardizing the burden over time. Conceptually, AUT integrates both the magnitude and duration of subthreshold BP, and TWA provides a time-normalized measure; both metrics have been used in perioperative studies to characterize intraoperative blood-pressure profiles and to examine associations with postoperative outcomes [18, 19].

### Primary outcome

The primary outcome was a composite of major complications, including 30-day mortality, reoperation, anastomotic leakage, pneumonia, reintubation, and prolonged ventilation exceeding 48 h [7].

### Secondary outcomes

The secondary outcomes assessed were anastomotic leakage and long-term survival. Survival duration was defined and calculated from the date of surgery to the last available data point as follows: (1) Patients receiving ongoing care at our institution: for patients who regularly visited the institution following surgery, survival was defined as the date of the most recent documented outpatient visit at the time of data collection. (2) Patients who died at our institution: for patients who died within the hospital following surgery, the survival duration was calculated from the date of surgery to that of death, as recorded in the medical records. (3) Patients transferred to other institutions: for patients transferred to other healthcare facilities postoperatively, survival data were collected through regular telephone follow-ups. Survival period was determined using the most recent updates received via these calls.

For patients lost to follow-up or who remained alive at the end of the study period, survival data were censored at the last follow-up.

### Statistical analysis

Continuous data were presented as medians with interquartile ranges, whereas categorical data were expressed as frequencies and percentages.

The analyses were predefined based on available literature and clinical relevance, with adjustments made for potential confounding variables. Multivariate logistic regression analysis was performed to investigate the association between IOH and outcomes, adjusting for age; sex; year of surgery; American Society of Anesthesiologists Physical Status (ASA-PS) classification; clinical tumor stage; body mass index (BMI); preoperative chemotherapy; preoperative radiotherapy; preoperative hemoglobin levels; preoperative estimated glomerular filtration rate; preoperative heart failure; presence of hypertension, diabetes, and chronic pulmonary disease; alcohol consumption habits; duration of surgery; intraoperative blood transfusion; intraoperative fluid balance; type of surgery (open or minimally invasive); and total intraoperative dose of phenylephrine and norepinephrine [11, 12, 20–22]. Smoking status was not entered as a separate covariate because of collinearity with chronic pulmonary disease. The multicollinearity of the regression model was assessed by the variance inflation factor. No combinations of covariates exhibited a variance inflation factor exceeding five; hence, all variables were included in the model. All analyses were adjusted for potential confounders unless otherwise specified. To assess the association between IOH and long-term survival rates, a Cox regression analysis was conducted. The covariates incorporated into this analysis were identical to those used in the logistic regression analysis. As this was an exploratory study, no corrections for multiple comparisons were made, and *P* > 0.05 was considered statistically significant.

All statistical analyses were performed using the R software, version 4.3.3 (R Foundation for Statistical Computing, Vienna, Austria).

### Sample size considerations

Prior to the study, statistical power calculations were not conducted, and the sample size was determined based on the data available.

According to methodological recommendations for logistic regression [23], a minimum of approximately 10 events per covariate is advised to ensure robust estimation and model validity. In our analysis, the ratio of anastomotic leakage events (*n *= 223) to the number of covariates (*n *= 21) substantially exceeded this threshold, thereby supporting the adequacy and reliability of the logistic regression model.

## Results

**Fig. 1:**
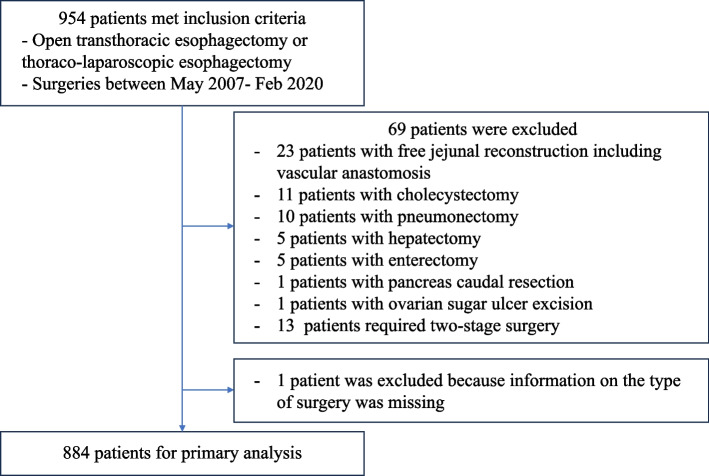
Flow diagram in the study

The final analysis included 884 patients, excluding individuals who underwent complex combined procedures, such as pneumonectomy or hepatectomy, or those with incomplete requisite data (Fig. 1). The median age of the participants was 66 years. The cohort was predominantly male, with female participants representing 18.6% of the population. The median BMI was 21.0 kg/m^2^. The majority of patients were classified as ASA-PS 2 (82.7%). Regarding cancer staging, stage 3 was the most prevalent, accounting for 45.8% of cases. A significant proportion of the cohort had undergone preoperative chemotherapy (58.3%), whereas a smaller subset underwent preoperative radiation therapy (13.5%). Additionally, comorbidities such as hypertension (34.4%), as well as smoking (79.8%), and alcohol abuse (77.5%) were observed. Of note, 18.7% of patients underwent open surgery, whereas 81.3% received minimally invasive procedures (Table 1).

**Table 1:** Patient characteristics and intraoperative data

Variables	Median or %
Age (years)	66 [60, 71]
Female (%)	162 (18.3%)
BMI (kg/m^2^)	21.0 [19.1, 23.0]
ASA-PS	
1	94 (10.6%)
2	731 (82.7%)
3	58 (6.6%)
4	1 (0.1%)
Stage classification	
1	122 (13.8%)
2	299 (33.8%)
3	405 (45.8%)
4	58 (6.6%)
Year of surgery	
2007	62 (7.0%)
2008	71 (8.0%)
2009	92 (10.4%)
2010	83 (9.4%)
2011	86 (9.7%)
2012	85 (9.6%)
2013	77 (8.7%)
2014	67 (7.6%)
2015	77 (8.7%)
2016	66 (7.5%)
2017	66 (7.5%)
2018	40 (4.5%)
2019	12 (1.4%)
Preoperative chemotherapy (%)	515 (58.3%)
Preoperative radiation therapy (%)	119 (13.5%)
Comorbidities	
Stroke (%)	43 (4.9%)
COPD (%)	115 (13.0%)
Heart failure (%)	26 (2.9%)
Hypertension (%)	304 (34.4%)
Diabetes (%)	122 (13.8%)
Renal disease (%)	146 (16.5%)
Liver disease (%)	133 (15.0%)
Anemia (%)	206 (23.3%)
Smoking (%)	705 (79.8%)
Alcohol abuse (%)	685 (77.5%)
Type of surgery	
Open surgery (%)	165 (18.7%)
Minimally invasive surgery (%)	719 (81.3%)
Duration of surgery (min)	495 [420, 570]
Cumulative vasopressor dosage	
Phenylephrine (mg)	0.3 [0.0, 0.8]
Norepinephrine (mg)	0.0 [0.0, 0.0]

Data are expressed as median [interquartile range] and number (%)

*Abbreviations*: *BMI* body mass index, *ASA-PS* American Society of Anesthesiologists physical status, *COPD* chronic obstructive pulmonary disease

### Major composite outcomes

The most prevalent postoperative complications were anastomotic leakage (25.2%) and pneumonia (23.4%). The 30-day mortality rate was 0.8%. Other notable complications included reintubation (12.4%) and prolonged ventilation exceeding 48 h (6.8%) (Table 2). Our analysis found no significant association between IOH and major composite outcomes across MBP and SBP thresholds. Adjusted odds ratios remained close to 1.00 across all thresholds, indicating minimal impact on major composite outcomes, such as pneumonia, prolonged ventilation, reintubation, or acute myocardial infarction (Table 3).

**Table 2:** Postoperative complications

Outcomes	*n* (%)
**Primary outcome**	
Composite of major complications	430 (48.6%)
30-day mortality	7 (0.8%)
Reoperation	60 (6.8%)
Anastomotic leakage	223 (25.2%)
Pneumonia	207 (23.4%)
Reintubation	110 (12.4%)
Prolonged ventilation exceeding 48 h	60 (6.8%)
**Others**	
Acute myocardial infarction	2 (0.2%)
Arterial fibrillation	77 (8.7%)
Arrhythmias other than arterial fibrillation	44 (5.0%)
Stroke	6 (0.7%)
Delirium	73 (8.3%)
Acute kidney injury	57 (6.4%)
Hemodialysis	16 (1.8%)
Pulmonary embolism	4 (0.5%)
Ileus	41 (4.6%)
Wound infection	121 (13.7%)

Data are expressed as number (%)

**Table 3:** Association between intraoperative hypotension and postoperative major composite outcome

Exposure	Adjusted odds ratio (95% CI)	*P* value
Time of MBP ^a^		
< 65 mmHg	1.00 (0.99, 1.02)	0.58
< 60 mmHg	1.01 (0.98, 1.03)	0.70
< 55 mmHg	1.00 (0.95, 1.06)	0.94
AUT of MBP ^b^		
< 65 mmHg	1.00 (0.99, 1.01)	0.80
< 60 mmHg	1.00 (0.98, 1.02)	0.91
< 55 mmHg	0.99 (0.94, 1.05)	0.82
TWA of MBP ^c^		
< 65 mmHg	1.00 (0.998, 1.002)	0.77
< 60 mmHg	1.00 (0.996, 1.004)	0.95
< 55 mmHg	1.00 (0.99, 1.01)	0.76
Time of SBP ^a^		
< 90 mmHg	1.01 (0.99, 1.04)	0.31
< 80 mmHg	1.01 (0.96, 1.07)	0.64
< 70 mmHg	1.02 (0.87, 1.21)	0.81
AUT of SBP ^b^		
< 90 mmHg	1.00 (0.99, 1.02)	0.48
< 80 mmHg	1.00 (0.97, 1.04)	0.91
< 70 mmHg	1.01 (0.91, 1.12)	0.93
TWA of SBP ^c^		
< 90 mmHg	1.00 (0.999, 1.003)	0.54
< 80 mmHg	1.00 (0.99, 1.01)	0.93
< 70 mmHg	1.00 (0.98, 1.02)	0.88

The odds ratio was estimated from a multivariate logistic regression analysis, adjusting for age, gender, year of surgery, American Society of Anesthesiology Physical Status classification, clinical tumor stage, body mass index, preoperative chemotherapy, preoperative radiotherapy, preoperative hemoglobin levels, preoperative estimated glomerular filtration rate, preoperative heart failure, presence of hypertension, diabetes, chronic pulmonary disease, alcohol consumption habits, duration of surgery, blood transfusion, fluid balance, type of surgery (open or minimally invasive), phenylephrine dosage, and norepinephrine dosage

*Abbreviations*: *CI* confidence interval, *AUT* area under the threshold, *TWA* time-weighted average, *MBP* mean blood pressure, *SBP* systolic blood pressure

^a^The adjusted odds ratio represents the value for every 10-min increase in the duration below the threshold

^b^The adjusted odds ratio represents the value for every 50 mmHg·minutes increase in AUT

^c^The adjusted odds ratio represents the value for every 1 mmHg increase in TWA

### Anastomotic leakage

Notably, IOH was associated with anastomotic leakage when MBP remained below 65 mmHg for extended periods. This relationship was quantified with an adjusted odds ratio of 1.02 per 10-min interval (95% confidence interval: 1.01–1.04, *P* = 0.01), demonstrating an elevated risk with prolonged hypotension (Table 4).

**Table 4:** Association between intraoperative hypotension and anastomotic leakage

Exposure	Adjusted odds ratio (95% CI)	*P* value
Time of MBP ^a^		
< 65 mmHg	1.02 (1.01, 1.04)	0.01*
< 60 mmHg	1.03 (1.00, 1.06)	0.03*
< 55 mmHg	1.05 (0.99, 1.12)	0.08
AUT of MBP ^b^		
< 65 mmHg	1.01 (1.00, 1.02)	0.04*
< 60 mmHg	1.02 (0.999, 1.048)	0.06
< 55 mmHg	1.04 (0.98, 1.10)	0.19
TWA of MBP ^c^		
< 65 mmHg	1.0018 (0.9999, 1.0038)	0.06
< 60 mmHg	1.003 (0.999, 1.007)	0.10
< 55 mmHg	1.005 (0.995, 1.015)	0.29
Time of SBP ^a^		
< 90 mmHg	1.02 (0.99, 1.04)	0.18
< 80 mmHg	1.01 (0.96, 1.07)	0.65
< 70 mmHg	1.10 (0.93, 1.30)	0.24
AUT of SBP ^b^		
< 90 mmHg	1.01 (0.99, 1.02)	0.32
< 80 mmHg	1.01 (0.98, 1.05)	0.44
< 70 mmHg	1.07 (0.97, 1.18)	0.19
TWA of SBP ^c^		
< 90 mmHg	1.001 (0.999, 1.003)	0.33
< 80 mmHg	1.002 (0.996, 1.008)	0.48
< 70 mmHg	1.01(0.99, 1.03)	0.23

The odds ratio was estimated from a multivariate logistic regression analysis, adjusting for age, gender, year of surgery, American Society of Anesthesiology Physical Status classification, clinical tumor stage, body mass index, preoperative chemotherapy, preoperative radiotherapy, preoperative hemoglobin levels, preoperative estimated glomerular filtration rate, preoperative heart failure, presence of hypertension, diabetes, chronic pulmonary disease, alcohol consumption habits, duration of surgery, blood transfusion, fluid balance, type of surgery (open or minimally invasive), phenylephrine dosage, and norepinephrine dosage

*Abbreviations:* *CI *confidence interval, *AUT* area under the threshold, *TWA* time-weighted average, *MBP* mean blood pressure, *SBP* systolic blood pressure

^a^The adjusted odds ratio represents the value for every 10-min increase in the duration below the threshold

^b^The adjusted odds ratio represents the value for every 50 mmHg·minutes increase in AUT

^c^The adjusted odds ratio represents the value for every 1 mmHg increase in TWA

*denotes a *P* value of less than 0.05

### Long-term survival

IOH did not significantly impact long-term survival, as indicated by hazard ratios of approximately 1.00 across all MBP and SBP thresholds. This suggests that, although IOH may influence immediate postoperative outcomes, its effect on long-term survival is minimal (Table 5).

**Table 5:** Association between intraoperative hypotension and long-term survival

Exposure	Hazard ratio (95% CI)	*P* value
Time of MBP ^a^		
< 65 mmHg	1.01 (0.99, 1.02)	0.39
< 60 mmHg	1.01 (0.99, 1.03)	0.32
< 55 mmHg	1.02 (0.98, 1.07)	0.34
AUT of MBP ^b^		
< 65 mmHg	1.004 (0.995, 1.013)	0.40
< 60 mmHg	1.01 (0.99, 1.03)	0.34
< 55 mmHg	1.02 (0.97, 1.06)	0.51
TWA of MBP ^c^		
< 65 mmHg	1.001 (0.999, 1.002)	0.48
< 60 mmHg	1.001 (0.998, 1.004)	0.42
< 55 mmHg	1.00 (0.99, 1.01)	0.64
Time of SBP ^a^		
< 90 mmHg	1.00 (0.98, 1.02)	0.90
< 80 mmHg	1.00 (0.96, 1.04)	0.96
< 70 mmHg	0.98 (0.88, 1.11)	0.79
AUT of SBP ^b^		
< 90 mmHg	1.00 (0.99, 1.01)	0.99
< 80 mmHg	1.00 (0.98, 1.02)	0.86
< 70 mmHg	1.00 (0.93, 1.07)	0.93
TWA of SBP ^c^		
< 90 mmHg	1.000 (0.999, 1.002)	0.94
< 80 mmHg	1.000 (0.995, 1.004)	0.85
< 70 mmHg	1.00 (0.99, 1.01)	0.93

The hazard ratio was estimated from a Cox regression analysis, adjusting for age, gender, year of surgery, American Society of Anesthesiology Physical Status classification, clinical tumor stage, body mass index, preoperative chemotherapy, preoperative radiotherapy, preoperative hemoglobin levels, preoperative estimated glomerular filtration rate, preoperative heart failure, presence of hypertension, diabetes, chronic pulmonary disease, alcohol consumption habits, duration of surgery, blood transfusion, fluid balance, type of surgery (open or minimally invasive), phenylephrine dosage, and norepinephrine dosage

*Abbreviations*: *CI* confidence interval, *AUT* area under the threshold, *TWA* time-weighted average, *MBP* mean blood pressure, *SBP* systolic blood pressure

^a^The hazard ratio represents the value for every 10-min increase in the duration below the threshold

^b^The hazard ratio represents the value for every 50 mmHg·minutes increase in AUT

^c^The hazard ratio represents the value for every 1 mmHg increase in TWA

## Discussion

This study, involving over 800 esophagectomy cases, revealed no significant association between IOH and postoperative composite complications or long-term survival. However, there was a significant association between IOH and anastomotic leakage. Considering the sample size, this study is the largest to examine the relationship between IOH and complications following esophagectomy.

The lack of an observed association between IOH and postoperative composite complications in this study may be attributed to the nature of the primary outcome, which was a composite measure. The composite outcome combined complications strongly associated with IOH and others less so, potentially diluting the observable effect of IOH on the overall outcomes. Additionally, although there are reports linking IOH to 30-day mortality in major surgeries [3, 24], the incidence of 30-day mortality within our composite outcomes was very low (7 cases, 0.8%), limiting the ability to assess its impact in this study.

Recent literature has suggested that IOH is associated with decreased long-term survival in older patients, potentially due to early postoperative complications, such as AKI and myocardial injury, or even through mechanisms promoting cancer cell proliferation [6]. However, no significant association was found between IOH and long-term prognosis in our study. This may be attributed to the predominant influence of factors such as cancer staging, chemotherapy, and radiation therapy on long-term survival rates, particularly in surgeries for esophageal cancer, overshadowing the impact of IOH.

We investigated the relationship between IOH and postoperative anastomotic leakage. Anastomotic leakage is influenced by multiple factors, including patient characteristics, comorbid conditions, and perfusion of the gastric conduit [11, 25]. Our research yielded a significant relationship between MBP during surgery and the incidence of anastomotic failure. Because MBP plays a key role in determining tissue perfusion pressure rather than SBP, this correlation is biologically plausible. Our analysis revealed that neither the AUT nor the TWA of the IOH were significantly associated with anastomotic leakage. However, the duration below the MBP threshold was significantly associated with these leakages. This suggests that even mild but prolonged episodes of low BP may impact tissue perfusion more significantly than do shorter episodes of more pronounced hypotension, potentially increasing the risk of anastomotic failure. There is limited literature exploring the relationship between IOH and anastomotic leakage, and the results of these studies are contradictory [13, 14]. One study, similar to ours, indicated a correlation between IOH and anastomotic leakage [13], whereas another study found no such relationship [14]. This discrepancy could be attributed to the different definitions of IOH employed. Unlike our study, which primarily focused on MBP, several studies concentrated on SBP. Given that perfusion of the gastric conduit is linked to anastomotic leakage [21], MBP provides a more relevant measure rather than SBP as a criterion for evaluating the association between IOH and complications.

Although a correlation has been observed between intraoperative MBP reduction and postoperative anastomotic leakage, it remains unclear whether management strategies aimed at avoiding IOH can effectively reduce the incidence of these leakages. The impact of avoiding IOH on postoperative complications has been examined in several randomized controlled trials. For instance, in non-cardiac surgery among older patients, a study that compared target MBP groups of 60–70 mmHg and 90–100 mmHg demonstrated a lower incidence of postoperative delirium in the group that maintained higher MBP levels [26]. Conversely, a study that divided non-cardiac surgery patients into groups with intraoperative target MBP > 60 mmHg or > 75 mmHg revealed no significant intergroup differences in the rates of postoperative AKI or acute myocardial injury [27]. Furthermore, a randomized controlled trial comparing a low hypotension avoidance strategy (MBP > 80 mmHg) with a lower threshold group (MBP > 60 mmHg) in non-cardiac surgeries revealed no significant difference in the incidence of major vascular complications postoperatively [28]. These findings suggest that, although some studies affirm that preventing hypotension can reduce postoperative complications, this is inconsistent across different types of complications or surgical settings. Therefore, prospective interventional studies are needed to ascertain whether the prevention of IOH can decrease the risk of anastomotic leakage and potentially guide future clinical protocols for surgical management and patient care.

In the present cohort, cumulative vasopressor use was not an independent determinant of postoperative morbidity, including anastomotic leakage. This finding is consistent with recent studies that similarly reported no significant association between vasopressor administration and an increased risk of anastomotic leakage [12, 29]. While the administration of excessive doses of vasoactive drugs has the potential to theoretically impair regional blood flow, appropriately titrated agents may preserve conduit perfusion and obviate fluid overload that promotes interstitial edema. The concept is supported by a laser-speckle contrast imaging study, which demonstrated that the administration of phenylephrine boluses for hypotension resulted in enhanced microcirculatory flux within the gastric conduit [30]. It is important to note, however, that hemodynamic management in our study—BP targets, choice and dose of vasopressor, and fluid administration—was not governed by a formal protocol but left to the attending anesthetist's discretion. Consequently, prospective investigations guided by a structured protocol will be imperative to elucidate the intricate interplay among IOH, vasopressor therapy, and fluid balance.

Our findings are constrained by some limitations. First, as this was a retrospective study, similar to other studies of this nature, identifying and fully controlling for all confounding factors proved challenging. Consequently, our findings may have been biased by both known and unknown confounders, albeit insufficiently characterized. This inherent limitation suggests that the results may not perfectly reflect the true effects studied. Second, hemodynamic exposure was confined to the intraoperative period, and we did not collect postoperative BP, cumulative fluid balance, or vasopressor dosing. Consequently, we cannot determine whether prolonged IOH directly impairs anastomotic perfusion or whether the observed association with leakage is mediated by subsequent postoperative hypotension and its management (e.g., vasopressor administration and fluid loading). Prospective studies that capture both intra- and postoperative hemodynamics—including BP, fluid balance, and vasopressor dose—are needed to clarify causality.

Second, our study focused exclusively on intraoperative BP and did not include an analysis of postoperative BP. While intraoperative hypotension is certainly critical, postoperative hypotension in the intensive care setting may also significantly influence postoperative complications. However, our study was not designed to assess these postoperative variables, which may limit the comprehensive understanding of the outcomes related to BP management during and after surgery.

## Conclusion

In conclusion, this study did not identify a significant association between IOH and the occurrence of complex postoperative complications or long-term outcomes. However, a significant association was observed between IOH and postoperative anastomotic leakage. Conducting a prospective interventional study is essential to determine whether preventing IOH can effectively reduce the incidence of postoperative anastomotic leakage.

## Data Availability

The datasets used and/or analyzed during the current study are available from the corresponding author on reasonable request.

## Abbreviations

AKI: Acute kidney injury
ASA-PS: American Society of Anesthesiology Physical Status
AUT: Area under the threshold
BMI: Body mass index
BP: Blood pressure
DBP: Diastolic blood pressure
IOH: Intraoperative hypotension
MBP: Mean blood pressure
TWA: Time-weighted average
